# Macrophages in obesity-related breast cancer: mechanistic insights and therapeutic opportunities

**DOI:** 10.1186/s13058-025-02155-x

**Published:** 2025-11-27

**Authors:** Huiyu Dong, Ming Zhou, Qin Sun, Yixing Ren, Lunkun Ma

**Affiliations:** 1 Nanomedicine Innovation Research and Development Transformation Institute, Affiliated Hospital of North Sichuan Medical College, Nanchong, 637000 People’s Republic of China; 2 Institute of Hepato-Biliary-Pancreatic-Intestinal Disease, Affiliated Hospital of North Sichuan Medical College, Nanchong, 637000 People’s Republic of China; 3 Department of Gastrointestinal Surgery, Affiliated Hospital of North Sichuan Medical College, Nanchong, 637000 People’s Republic of China; 4 School of Management, Key Laboratory of Digital-Intelligent Disease Surveillance and Health Governance, North Sichuan Medical College, Nanchong, People’s Republic of China

**Keywords:** Obesity-related breast cancer, Adipose tissue, Macrophages, Tumor microenvironment

## Abstract

Obesity has emerged as a global public health crisis, significantly increasing susceptibility to malignancies, including breast cancer. In the context of Obesity-Related Breast Cancer (ORBC), the abundant adipose tissue in the mammary gland profoundly reshapes the tumor microenvironment (TME). Whereas obesity drives chronic, low-grade inflammation through pro-inflammatory M1 adipose tissue macrophages (ATMs), tumor-associated macrophages (TAMs) acquire a predominantly immunosuppressive M2 phenotype that promotes angiogenesis, immune evasion, and treatment resistance. This review examines macrophage ontogeny, polarization, and functional heterogeneity in ORBC, highlighting how obesity-induced cytokines (e.g. TNF-α, IL-6), reactive oxygen species, and metabolic reprogramming collectively reshape the TME to foster tumor initiation, progression, and metastasis. We further evaluate emerging therapeutic strategies—such as CSF-1R inhibitors, PD-1/PD-L1 checkpoint blockade, and CAR-macrophage immunotherapies—that target macrophages recruitment, polarization, and immunometabolic pathways in ORBC.

## Introduction

Obesity has become a global pandemic With the World Obesity Atlas 2024 projecting that over 54% of the world's adult population will be overweight or obese by 2035 based on BMI criteria [1]. Once prevalent mainly in developed nations, this metabolic disorder is now increasingly widespread in developing countries [2]. Parallel to this trend, global cancer incidence escalates, with projections exceeding 35 million new cases annually by 2050, among which Obesity-Related Breast Cancer (ORBC) represents a growing clinical challenge.

Epidemiological evidence firmly establishes obesity as a significant risk factor for breast cancer, particularly in postmenopausal populations [3]. The ORBC involves multiple pathophysiological mechanisms, including chronic low-grade inflammation, metabolic dysregulation (e.g. hyperinsulinaemia, dyslipidaemia), and adipokine dysregulation. These factors collectively foster a pro-tumorigenic microenvironment that promotes immune evasion, angiogenesis, and epithelial-mesenchymal transition(EMT) [4].

Within this altered microenvironment, macrophages serve as pivotal immunomodulators with context-dependent functions in ORBC progression. While classically activated M1 macrophages exhibit tumoricidal activity, obesity promotes polarization toward an M2 tumor-associated macrophages (TAM) phenotype. These TAMs secrete immunosuppressive cytokines (IL-10, TGF-β) and matrix-remodeling enzymes (MMPs), ultimately driving metastasis and therapeutic resistance in breast cancer [5]. Building upon the central findings of this review, we summarize the regulatory mechanisms and challenges of macrophages in ORBC.

## Advances in macrophages biology

### The development and heterogeneity of macrophages

As innate immune sentinels, macrophages play an important role not only in obesity and breast cancer alone, but also in ORBC. Macrophages are central participants in both innate (non-specific immune) and adaptive immune (specific/cellular immune) responses and are key cells in the inflammatory response by phagocytosis and neutralization of cellular debris and pathogens such as bacteria and viruses, and activation of other immune cells that enable the body's tissues to function [6].

Macrophages primarily originate from two major developmental pathways. During embryogenesis, progenitors from the yolk sac and fetal liver give rise to tissue-resident macrophages, such as microglia, Kupffer cells, and alveolar macrophages, which are maintained largely through self-renewal independent of circulating monocytes. In contrast, during adulthood, bone marrow–derived hematopoietic stem cells generate circulating monocytes that can differentiate into macrophages upon recruitment to tissues, particularly under conditions of inflammation, infection, or cancer [7, 8]. This dual ontogeny underlies the remarkable heterogeneity of macrophage populations across tissues and disease contexts, highlighting their plasticity and functional diversity.

**Fig. 1:**
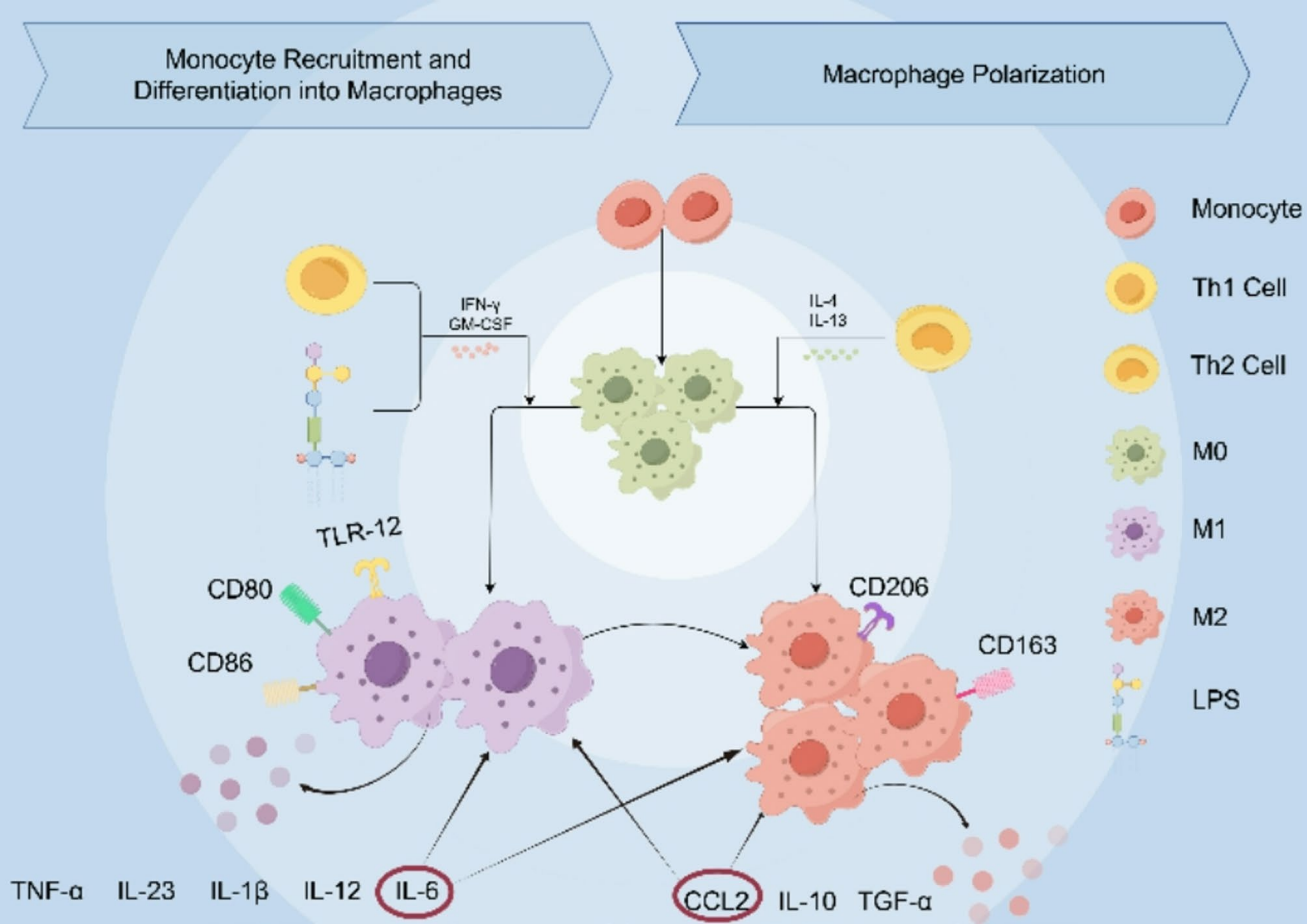
Monocyte Differentiation and Macrophage Polarization Monocytes differentiate into M0 macrophages and polarize into pro-inflammatory M1 (CD80, CD8…) or anti-inflammatory M2 (CD206, CD163…) phenotypes under cytokine stimulation. M1 macrophages secrete IL-6, IL-12, and TNF-α, etc., while M2 macrophages produce IL-10, CCL2, and TGF-α, etc. During acute inflammation and obesity, CCL2 promotes macrophage polarization toward the M1 phenotype. During acute inflammation and early obesity, IL-6 promotes macrophage polarization toward the M1 phenotype, whereas in late-stage obesity it promotes polarization toward the M2 phenotype. Within the tumor microenvironment, both CCL2 and IL-6 promote macrophage polarization toward the M2 phenotype

The current widely accepted classification of macrophages can be divided into two major groups: M1(classically activated macrophages) and M2 (alternatively activated macrophages) [9] where M2 can be divided into M2a, M2b, M2c, M2d, according to their functions and M2d is closely related to tumor progression [10]. when Lipopolysaccharide (LPS) activates STAT1 transcriptional regulators alone or in combination with Th1 cytokines such as IFN-γ and GM-CSF, the macrophages are polarized into M1 type, producing cytokines such as IL-1β, IL-6, IL-12, IL-23 and TNF-α, which are involved in host immune defense responses against various bacteria, protozoa and viruses, exerting pro-inflammatory and anti-tumor effects, and leading to tissue damage(Fig. 1); when Th2 and other cytokines such as IL-4 and IL-13 activate STAT3 and STAT6 transcriptional regulators, macrophages are polarized into the M2 type, producing cytokines such as IL-10 and TGF-β or pro-fibrotic mediators, playing pro-inflammatory, pro-tumorigenic, and pro-tissue repair roles [10–12]. Additionally, Both CCL2 and IL-6 exhibit context-dependent roles in macrophage polarization. In acute inflammation, IL-6 amplifies pro-inflammatory signaling and promotes M1 polarization [13], while CCL2 recruits CCR2⁺ monocytes that predominantly differentiate into M1 macrophages [14]. In obesity, IL-6 elevation initially drives M1-like adipose tissue macrophages (ATMs), contributing to insulin resistance [15], but sustained IL-6/STAT3 activation favors M2-like phenotypes with immunosuppressive features [16]. Similarly, CCL2-mediated monocyte recruitment exacerbates adipose inflammation via M1-like ATMs [17]. In the tumor microenvironment, however, both IL-6 and CCL2 predominantly support M2-like TAM polarization, thereby fostering angiogenesis, immune suppression, and tumor progression [18, 19].

Although classical M1 and M2 polarization is readily induced in vitro by strong, opposing stimuli. Sica and Mantovani emphasized this point, arguing that macrophage identity in pathological and physiological settings is best understood as a dynamic spectrum rather than fixed classes. In vivo, macrophages integrate a complex and changing milieu of cytokines, metabolic cues and spatial signals, resulting in highly plastic, context-dependent intermediate or transient phenotypes that often co-express markers and functions traditionally ascribed to both “M1-like” and “M2-like” phenotypes [20]. An increasing number of recent studies define macrophage phenotypes as transient and dynamic, rather than adhering to the canonical M1/M2 classification(e.g. LAMs) [21]. Importantly for breast cancer, single-cell and spatial profiling studies have revealed multiple tumor-associated macrophages (TAM) states with distinct transcriptional signatures, spatial distributions and functional associations that cannot be fully captured by the M1/M2 dichotomy, underscoring the need for spectrum- based or function-based definitions when linking macrophage phenotypes to tumor behavior and therapy response [22].

### The functional diversity of macrophages

**Fig. 2:**
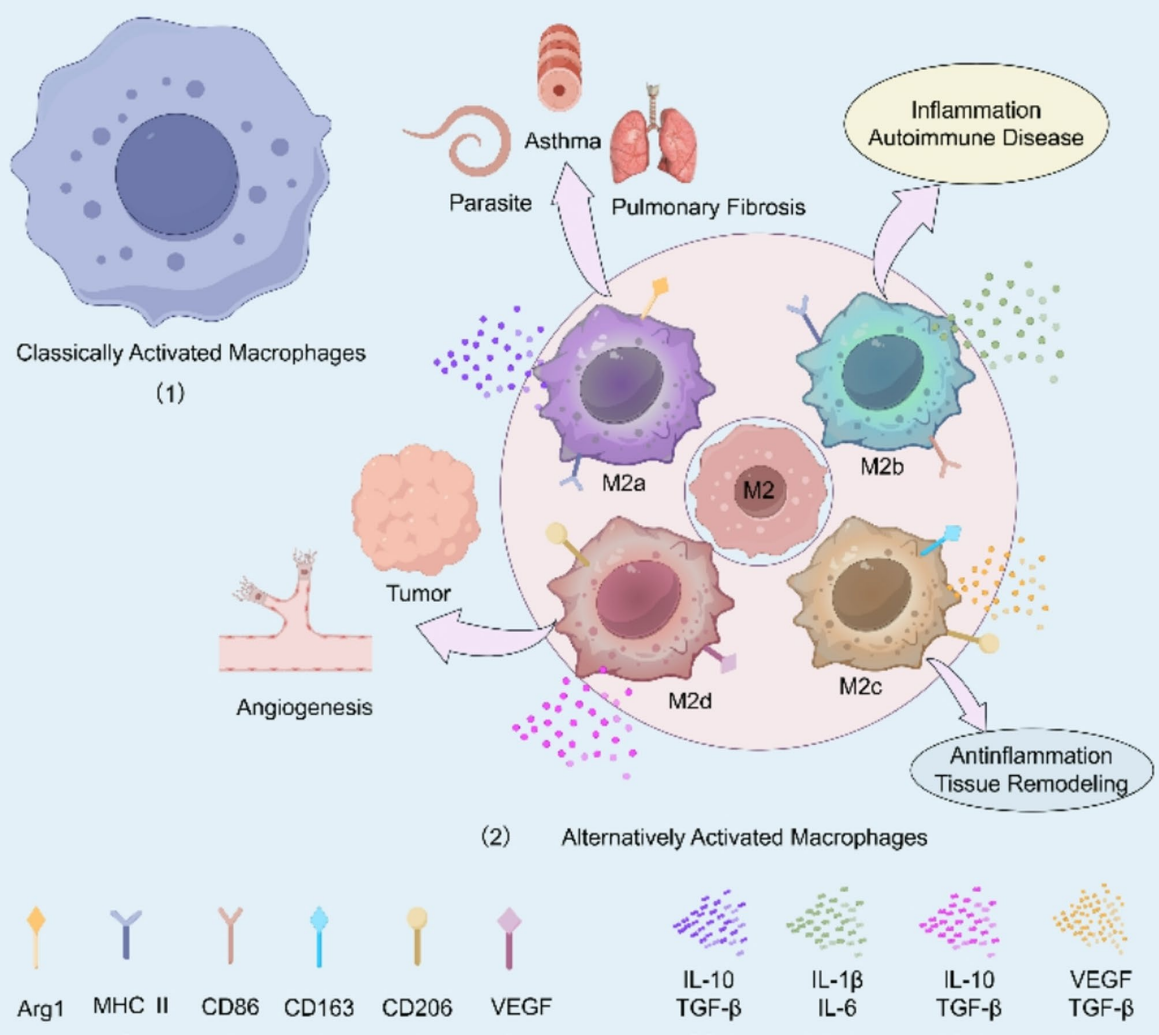
Classification of Macrophages and Subtypes: Macrophages are categorized into Classically Activated Macrophages(M1) and alternatively activated Macrophages(M2).there are four subtypes of M2 (M2a, M2b, M2c, M2d), each induced by distinct stimuli (e.g. IL-4, immune complexes, IL-10, and tumor factors, etc.) and associated with unique surface markers and cytokine profiles(Only Some of the cytokines and surface markers are shown in the graph).M1 macrophages contribute to pro-inflammatory responses and antitumor immunity, while M2 subtypes are involved in tissue remodeling, immunosuppression, and tumor progression. M2a expresses MHC-Ⅱ and Arg1, etc. M2b is marked by CD86 and MHC-II, etc. M2c by CD163 and CD206, etc. and M2d by CD206 and VEGF, etc. These subsets highlight the complexity and plasticity of macrophages in inflammation and cancer biology

The functions of macrophages vary depending on their origin and the tissues in which they are found. Macrophages have three main roles: phagocytosis, antigen presentation, and immunomodulation [23]. M1 is mostly associated with coronary heart disease and chronic inflammatory diseases such as inflammatory bowel disease and rheumatoid arthritis, as well as autoimmune diseases such as autoimmune hepatitis and Crohn's disease, while M2 is mainly associated with fibrotic diseases and wound healing [11], and it has been shown to play an important role in some allergies and asthma [24]. For example, in the process of tissue injury and repair, there are different types of macrophages, such as pro-inflammatory macrophages, pro-fibrotic macrophages, anti-inflammatory macrophages, etc [25]. The surface markers of the two are different, the surface markers of M1 are mainly iNOS (inducible nitric oxide synthase) 、CD80、CD86、TNF-α, the surface markers of M2 are mainly Arg1 (Arginase 1),CD163,CD206 [26] (Fig. 2). Phenotypes of predominantly occupying macrophages differ in different pathological types. For example, M1-M2 or M2 macrophages, which are predominantly expressed in some cancers, predominantly express M2 types in allergic reactions and M1 types in obese adipose tissue [20]. Thus, it can be seen that macrophages have significant heterogeneity due to temporal and spatial distribution [9], as well as specific phenotypes, which is likely to be a result of the corresponding microenvironment of the disease and the cellular interactions in the course of the evolution of the disease.

## Macrophages in obesity and breast cancer

### Adipose tissue dysfunction and macrophage polarization in obesity

Adipose tissue functions not only as an efficient energy reservoir but also as a dynamic endocrine organ [27]. Based on anatomical location, adipose tissue is categorized into subcutaneous adipose tissue (SAT) and visceral adipose tissue (VAT) [28]. SAT lies between the skin and muscle and comprises approximately 80% of total body fat. It serves as a "metabolic buffer pool" that stores excess energy. VAT, on the other hand, surrounds internal organs within the abdominal cavity, accounts for 10–20% of total fat, and releases large quantities of free fatty acids (FFA), contributing to insulin resistance and hepatic steatosis [29].

Functionally, adipose tissue is further divided into white, brown, and beige adipose tissue. White adipose tissue (WAT), the predominant form in both SAT and VAT, is primarily responsible for energy storage [30]. Brown adipose tissue (BAT), found mainly in the interscapular region and neck of neonates and small mammals, dissipates energy via heat generation. Beige fat, residing within WAT depots, can acquire thermogenic characteristics similar to BAT under certain stimuli such as cold exposure [31]. Among these, WAT plays a pivotal role in the pathophysiological responses associated with obesity.

Obesity, defined by the World Health Organization as an abnormal or excessive fat accumulation that may impair health, is typically diagnosed using body mass index (BMI), calculated as weight (kg)/height^2^ (m^2^). However, contemporary perspectives advocate a multidimensional and individualized assessment of obesity. Recent literature proposes a distinction between "clinical obesity"—characterized by systemic dysfunctions—and "preclinical obesity, " which represents excess adiposity with preserved organ function but elevated risk for various diseases, including type 2 diabetes, cardiovascular diseases, and certain cancers [33].

A hallmark of obesity is chronic low-grade inflammation, primarily initiated by dysfunctional adipocytes secreting chemokines such as CCL2, which drive the infiltration of immune cells—most notably macrophages [34, 35]. This infiltration is strongly correlated with both adipocyte size and BMI, and these adipose tissue macrophages (ATMs) have been identified as key producers of proinflammatory cytokines like TNF-α [36]. In both diet- and genetically induced obese mouse models, macrophage accumulation is most pronounced in VAT, particularly within crown-like structures (CLS), and to a lesser extent in SAT [37]. Gene expression analyses in obese mice and humans confirm a marked upregulation of macrophage-specific and inflammatory genes, especially in WAT [38]. These macrophages form CLS around necrotic adipocytes, contributing to persistent inflammation and metabolic disruption [39].

ATMs, the most abundant immune cell type in adipose tissue, can be either tissue-resident (embryonic origin) or derived from circulating monocytes [40, 41]. In lean individuals, ATMs predominantly exhibit an M2 anti-inflammatory phenotype that supports tissue homeostasis and lipid metabolism [42]. In contrast, obesity induces a phenotypic switch toward the pro-inflammatory M1 subtype, characterized by the release of TNF-α, IL-6, and IL-1β, which exacerbates adipose inflammation and contributes to systemic insulin resistance [43, 44]. ATM infiltration may increase from 4 to 50% in obese adipose tissue [45], and though their effects are largely restricted to adipose depots, they significantly influence systemic metabolic health.

In addition to M1/M2 phenotypes, emerging ATM subsets such as metabolically activated macrophages (MMe) and lipid-associated macrophages (LAM) have been identified [35]. MMe were first described as an obesity-induced ATM state in adipose depots driven by saturated free fatty acids and NOX2-dependent metabolic activation that secretes IL-6 and promotes cancer stem-like properties via GP130/STAT3 signaling [46]. while LAM is described a monocyte-derived, STAB1⁺/TREM2 high macrophage subset localized to tumor-adipose junctions (and inducible by cancer-associated fibroblast signals) that is enriched for FABPs, lipid catabolism genes and phagocytic programs and that mediates immune suppression and resistance to checkpoint blockade in breast cancer models [21].

Importantly, both MMe and LAM do not fit cleanly into the classical M1/M2 paradigm. But at the molecular level, the two programs share a substantive intersection: both MMe and LAM express elevated lipid-metabolism modules (FABPs, LPL, APOC/APOE family members) and stress-response genes, reflecting adaptation to free fatty acids and other lipid cues in obese adipose/tumor niches [47].so taken together, these data favor a model in which MMe and LAM represent related, lipid-programmed macrophage states within a continuous activation landscape rather than wholly distinct, invariant lineages.

### Macrophage subtypes in breast cancer via single cell RNA-seq

Recent single-cell and spatial transcriptomic studies have shown that tumor-associated macrophages (TAMs) in human breast cancers do not form a simple M1/M2 dichotomy but rather occupy multiple transcriptionally and spatially distinct states with specialized functions [48]. Across independent atlases, recurrent TAM states have been recovered-for example, complement-rich C1QC⁺/TREM2⁺ immunoregulatory macrophages, SPP1⁺ (osteopontin-expressing) matrix/lipid-associated populations linked to ECM remodeling and metastasis, FCN1⁺/monocyte-like inflammatory cells, and CCL18⁺ terminal immunosuppressive clusters-each enriched for different pathway programs and occupying distinct microanatomical niches [49]. Spatial mapping further demonstrates that SPP1⁺ and other lipid-/matrix-associated TAMs preferentially localize to peritumoral stromal/ECM niches and correlate with aggressive histology and poorer clinical outcome in multiple cohorts, whereas tissue-resident FOLR2⁺/LYVE1⁺ macrophage populations characteristic of normal mammary gland are depleted or transcriptionally reprogrammed in tumors [50].

Large, integrative single-cell studies focused on breast cancer have extended and refined this taxonomy: Zhang et al. constructed a breast-cancer TAM atlas that delineates seven transcriptional TAM subtypes (e.g. TUBA1B⁺, C1QC⁺, VCAN⁺, IL1B⁺, SLC40A1⁺, APOC1⁺, CXCL11⁺) whose relative abundance varies by molecular subtype and stage, implying functional specialization and dynamic remodeling within the TME [51]. Similarly, Gao et al. used a very large single-cell compendium to reveal clinically relevant macrophages and stromal subpopulations (including macrophage subsets such as CKB⁺ cells) that associate with genomic features, therapeutic sensitivity and outcome, underscoring that discrete TAM states-not a single “M2” program-drive immune suppression and therapy resistance in particular patient subsets [52]. The discovery of these subtypes enables us to delve deeper into the underlying mechanisms of ORBC.

### Macrophage-mediated immune remodeling in obesity-related breast cancer

Obesity also significantly alters the immune microenvironment of breast tissue, particularly through enhanced macrophages activity. Breast cancer is the most prevalent malignancy among women globally and remains a leading cause of cancer-related deaths. Histologically, breast cancer is classified into non-invasive (e.g. ductal carcinoma in situ) and invasive types (e.g. invasive lobular carcinoma, ductal carcinoma, mucinous carcinoma) [53]. Molecular subtyping based on gene expression—such as Luminal A, Luminal B, HER2-enriched, and basal-like subtypes (PAM50)—further refines diagnosis and therapeutic strategies [54], while newer classifications based on metabolic or immunological profiling are under active investigation [55].

The tumor microenvironment (TME) is a complex ecosystem comprising of immune cells, fibroblasts, adipocytes, mesenchymal stem cells, and extracellular matrix components [12, 56]. Among immune cells, tumor-associated macrophages (TAMs)—marked by CD68, CD163, CD204, and CD206—are key players [57]. TAMs may derive from either tissue-resident macrophages or peripheral sources such as the bone marrow and spleen [58]. In ORBC, TAM infiltration is linked to enhanced tumor aggressiveness, metastasis, and poor prognosis [59].

TAMs can polarize toward M1 or M2 phenotypes, although in breast cancer, M2 macrophages are typically more abundant [60]. Clinical studies report a significant reduction in M1 macrophages and an increase in M2 macrophages in the peripheral blood of breast cancer patients [61]. In vitro assays reveal that M2 macrophage-conditioned media enhances breast cancer cell migration, whereas M1 macrophages do not exert this effect [62]. The predominance of M2-type TAMs may be driven by oncogenic mechanisms such as SENP3 deficiency, which leads to increased Akt1 SUMOylation and promotes M2 polarization [63].

Recent findings suggest that TAMs undergo metabolic reprogramming, shifting toward glycolysis, fatty acid synthesis, and altered nitrogen metabolism [64]. These metabolic alterations are induced by TME-derived signals, including cytokines and metabolites secreted by tumor, stromal, and immune cells [65]. TAM-derived metabolites, in turn, perpetuate an immunosuppressive TME conducive to tumor progression.

## The dual role of macrophages in obesity-related breast cancer

Obesity, as an important risk factor behind the increased incidence of breast cancer, has produced an undeniably important contribution to ORBC. There is no strict definition of ORBC, which we consider to be a breast cancer that arises in the context of obesity and is caused directly or indirectly by obesity. Macrophages facilitate tumor immune escape through multiple mechanisms. Obesity promotes the functional enhancement of M2 macrophages (such as upregulation of PD-1 expression), thereby leading to angiogenesis, immunosuppression, and the promotion of tumor growth and metastasis [66].

### M2 in obesity-related breast cancer

#### M2 promotes immune evasion and tumor progression

M2 macrophages play a central role in promoting immunosuppression and angiogenesis within the breast tumor microenvironment. These macrophages, often polarized under the influence of tumor-derived and adipose-derived signals, secrete immunosuppressive cytokines such as IL-10, IL-8, and TGF-β, which contribute to the upregulation of immunosuppressive gene expression and the inhibition of antitumor immune responses [67, 68]. Moreover, A research demonstrates that in human breast tissue, obesity correlates with an increased proportion of M2-biased macrophages, and obesity further elevates the number of M2-biased macrophages within the tissue [69]. Therefore, this may also serve as evidence that obesity exacerbates the effects of M2 macrophages in the tumor microenvironment.

**Fig. 3:**
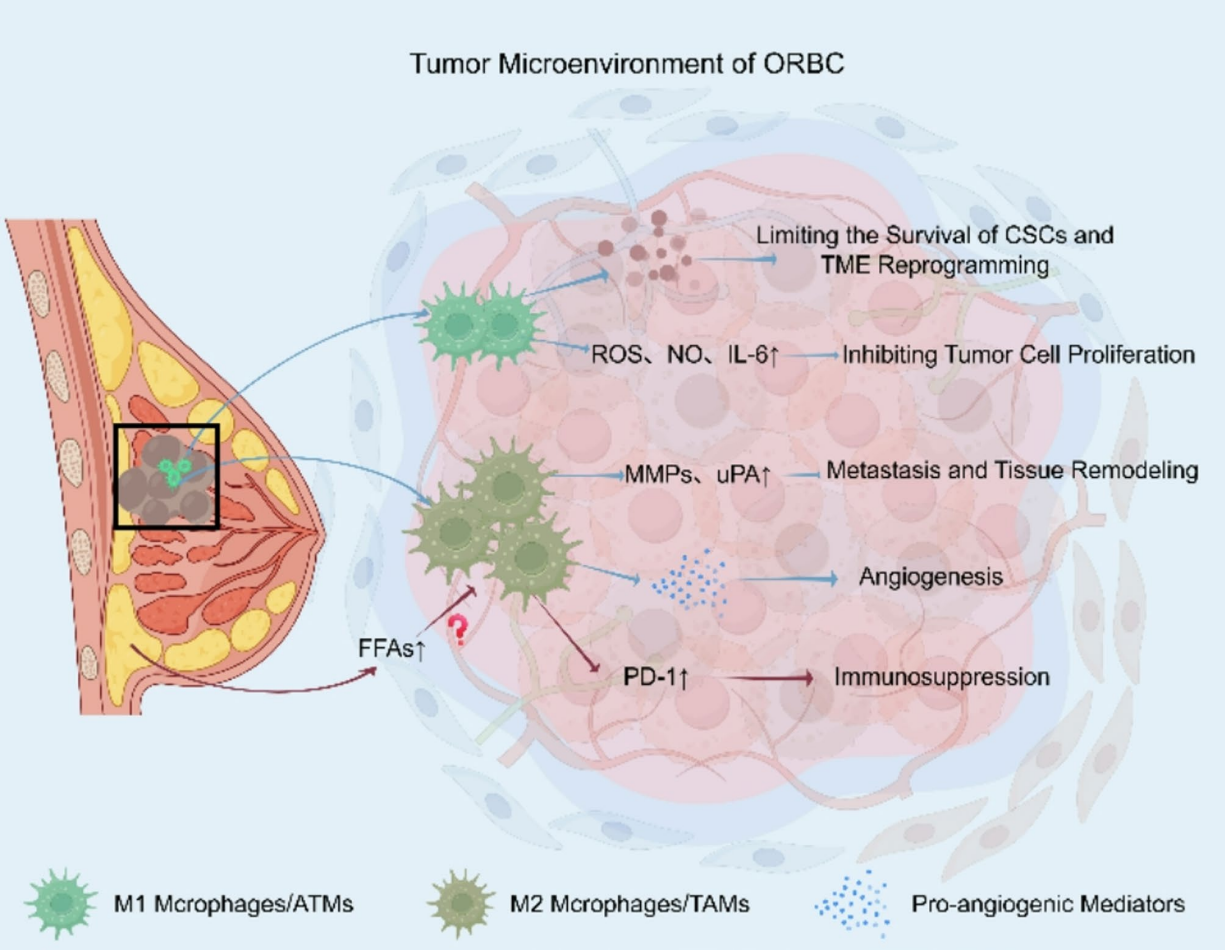
Distinct Roles of M1 and M2 Macrophages in the Breast Tumor Microenvironment: M1 macrophages (ATMs) promote antitumor immunity through the release of ROS, NO, and IL-6, thereby inhibiting tumor cell proliferation, reprogramming the TME, and limiting the survival of cancer stem cells (CSCs). Meanwhile, M2 macrophages (TAMs), enriched in obesity, are activated and promote immunosuppression via PD-1 upregulation. They also facilitate angiogenesis, metastasis, and tissue remodeling through the secretion of pro-angiogenic mediators, MMPs and uPA, contributing to tumor progression

Moreover, M2 macrophages respond to the metabolic shifts within the tumor microenvironment, particularly the accumulation of lactic acid resulting from the Warburg effect, in which tumor cells preferentially utilize anaerobic glycolysis even under normoxic conditions [70]. The resulting acidic environment impairs the function of immune effector cells such as natural killer (NK) cells and cytotoxic T lymphocytes, while simultaneously promoting the expression of immunosuppressive mediators by M2 macrophages [71]. Obesity has been shown to enhance PD-1/PD-L1 signaling in the tumor microenvironment, including increased PD-1 expression on macrophages, thereby suppressing antitumor immune responses [66]. Additionally, there is currently no direct evidence indicating whether the large amount of free fatty acids (FFAs) produced by obesity exacerbates the aforementioned effect (Fig. 3). Additionally, obesity leads to the accumulation of free fatty acids (FFAs). We hypothesize that high levels of FFAs may exacerbate the aforementioned effects; however, direct evidence for this remains lacking at present. Collectively, these findings underscore the multifaceted role of M2 macrophages in shaping an immunosuppressive and pro-angiogenic niche that fosters breast cancer progression, particularly in the context of obesity.

#### TAMs(M2-like phenotype) promote angiogenesis and metastasis in ORBC

Angiogenesis serves as a critical bridge for the metastasis and dissemination of tumor cells. Tumor-associated macrophages (TAMs) exhibiting the M2 phenotype constitute a heterogeneous population shaped by the tumor microenvironment, functioning as key regulators of tumor angiogenesis. TAMs secrete a broad range of cytokines and growth factors, including vascular endothelial growth factor (VEGF), transforming growth factor-β (TGF-β), epidermal growth factor (EGF), platelet-derived growth factor (PDGF), and CCL18, which collectively stimulate endothelial cell proliferation and the formation of new vasculature within the tumor microenvironment [72]. Furthermore, adipocyte-derived leptin in obese individuals has been shown to upregulate the expression of these angiogenic mediators in macrophages, further enhancing neovascularization [73].

Once angiogenesis is initiated, TAMs facilitate tumor invasion and tissue remodeling by producing matrix-degrading enzymes such as matrix metalloproteinases (MMPs) and urokinase-type plasminogen activator (uPA), which degrade the extracellular matrix (ECM) and basement membranes [74]. This degradation enables cancer cells to invade adjacent tissues and gain access to blood vessels, ultimately enhancing their potential for intravasation and distant metastasis [75]. Importantly, the neovasculature formed under the influence of TAMs is often disorganized and leaky, further aiding tumor cell migration into the circulatory system.

In addition to directly promoting vessel formation and matrix remodeling, TAMs contribute to pre-metastatic niche formation by secreting cytokines and chemokines that recruit immunosuppressive and pro-metastatic cells to distant organs, thus priming these sites for secondary tumor colonization [12]. These multifaceted functions underscore the central role of TAMs in both the initiation of angiogenesis and the facilitation of metastatic dissemination, especially in the pro-inflammatory and lipid-rich context of obesity.

### M1 in obesity-related breast cancer

#### Antitumor role of M1 macrophages in obesity-related breast cancer

M1 macrophages, also known as classically activated macrophages, exhibit prominent antitumor properties, largely mediated through their pro-inflammatory activity. Upon stimulation by IFN-γ and lipopolysaccharide (LPS), M1 macrophages secrete high levels of inflammatory cytokines such as TNF-α, IL-1β, IL-6, and IL-12, as well as reactive oxygen species (ROS) and nitric oxide (NO), which contribute to the direct killing of tumor cells and the activation of Th1-type immune responses [76]. This pro-inflammatory phenotype not only hinders tumor cell survival and proliferation but also inhibits tumor angiogenesis and immune evasion mechanisms. In contrast to the tumor-promoting M2 phenotype, the presence of M1 macrophages in the tumor microenvironment (TME) has been associated with improved clinical outcomes and reduced tumor progression [77].

Furthermore, in the context of obesity—a known risk factor for postmenopausal breast cancer—chronic low-grade inflammation induced by dysfunctional adipose tissue fosters a tumor-permissive TME. Yet, the skewing of macrophages polarization toward the M2 phenotype in obese individuals highlights the therapeutic potential of reprogramming tumor-associated macrophages (TAMs) toward the M1 phenotype [78–80]. TLR signaling plays a central role in bridging innate immune responses with tumor suppression by promoting M1 polarization and sustaining inflammatory responses against cancer cells [76]. Oxidative stress, although elevated in obesity, when localized and regulated via M1 macrophages activation, may enhance anti-tumor immunity through enhanced antigen presentation and cytotoxicity [81, 82].

These insights emphasize the importance of enhancing or maintaining M1 polarization in the breast tumor microenvironment, particularly in ORBC, where inflammation and immune dysfunction coexist.

#### Tumor microenvironment reprogramming and cancer stem cell maintenance

While M2-type tumor-associated macrophages (TAMs) are well recognized for their immunosuppressive and tumor-promoting functions in breast cancer, M1 macrophages—classically activated by IFN-γ and microbial stimuli—exert contrasting effects by promoting antitumor immunity and reprogramming the tumor microenvironment (TME) toward a more hostile landscape for cancer progression [83, 84]. Under physiological and early tumorigenic conditions, M1 macrophages secrete pro-inflammatory cytokines such as TNF-α, IL-12, and IL-1β, which not only recruit cytotoxic immune cells but also inhibit cancer stem cell (CSC) maintenance by disrupting the stem-supportive niche [85].

M1 macrophages are capable of remodeling the TME by enhancing oxidative stress and immune surveillance. Through the release of reactive oxygen species (ROS) and nitric oxide (NO), classically activated macrophages can impose genotoxic and cytotoxic stress on tumor cells and thereby limit the survival of cancer stem-like cells (CSCs) in some experimental settings, which are otherwise relatively resistant to oxidative insults [86]. In contrast to M2 macrophages that commonly support epithelial–mesenchymal transition (EMT) and metastasis, M1 macrophages have been reported to antagonize EMT-related transcriptional programs and reduce CSC plasticity in model systems — effects that may be mediated, at least in part, via modulation of Notch and WNT/β-catenin signaling pathways [87].

In addition, M1-associated inflammatory signals can increase the metabolic vulnerability of CSCs. Proinflammatory cytokines (for example, IL-12) promote antitumor immunity and can reprogram tumor and stromal signaling networks that influence redox balance and nutrient-sensing pathways; however, direct evidence for a linear pathway in which IL-12 from M1 macrophages inhibits PI3K/AKT/mTOR leading to NRF2 suppression in CSCs remains limited and should be presented as a plausible mechanism requiring further validation [88]. Under hypoxic conditions, where CSCs commonly upregulate glycolysis via HIF-1α to survive nutrient-deprived niches, inflammatory mediators associated with M1 activation can alter HIF-1α signaling and metabolic programs in both myeloid and tumor cells, potentially counteracting some hypoxia-driven survival adaptations [89]. Moreover, M1-derived extracellular vesicles/exosomes enriched in immune-stimulatory RNAs and miRNAs have been reported in several contexts to modulate tumor–stroma communication, relieve immune suppression, and in some models inhibit angiogenesis or reprogram pro-tumor TAMs — although the specific effects on CSC-supportive extracellular matrix formation are still being elucidated [90].

These functions are particularly relevant in the context of ORBC, where chronic low-grade inflammation and adipose/tumor metabolic reprogramming favor accumulation of M2-like macrophages and enrichment of CSC phenotypes; shifting the balance toward M1 activation is therefore a rational therapeutic hypothesis, but the precise molecular pathways and causal links require further experimental confirmation in ORBC models [46].

## Therapeutic strategies targeting macrophages in obesity-related breast cancer (ORBC)

In ORBC, macrophages, especially tumor-associated macrophages (TAMs), play a critical role in tumor progression, immune evasion, and therapy resistance. As a result, targeting TAMs has become a promising strategy in breast cancer therapy. Although immune checkpoint blockade (ICB) has demonstrated efficacy in some cancers, its effectiveness in ORBC is limited due to immune resistance mechanisms mediated by TAMs [91, 92]. In fact, a study have described an “obesity paradox,”whereby excess body weight is paradoxically associated with improved responses to immunotherapy across multiple cancer types. The specific mechanism remains to be further explored [93]. Notably, upregulation of immunosuppressive pathways such as the CSF1 axis, driven by MORF4L2, enhances M2 polarization and suppresses anti-tumor immunity [94]. This section outlines current and emerging macrophage-targeted strategies in ORBC, structured according to functional mechanisms. (Table 1).

**Table 1:** Innovative Strategies Targeting TAMs in Obesity-Related Breast Cancer

Example Agents/Tools	Strategy	Mechanism	Clinical Stage	References
CCL2 inhibitors	Inhibiting chemokine signaling	Blocks macrophages recruitment	Phase Ⅰ	[95]
Clodronate	Depleting macrophages	Reduces total macrophages population	Phase III(Mainly used for bone metastasis of breast cancer)	[96]
CSF1R inhibitors; PI3K-γ inhibitors	Reprogramming TAM phenotypes	Promotes M1 polarization, enhances immune responses	Phase Ⅰ/Ⅱ	[73, 99]
Anti-PD-L1 antibodies	Immune checkpoint blockade	Restores T cell function, reverses TAM suppression	Phase Ⅰ/Ⅱ in ORBC	[97, 98]
Anti-IL-10; Anti-TGF-β antibodies	Cytokine neutralization	Blocks immunosuppressive signals from TAMs	Preclinical; Phase Ⅰ/Ⅰb	[100]
MOF-NPs; Ferroptosis-inducing NPs	Nanobiotechnology	Targeted delivery and phenotypic modulation of TAMs	Preclinical	[101, 102]
CAR-M	CAR-M therapy	Enhances phagocytosis and antitumor inflammation	Phase Ⅰ, first-in-human trial(HER2⁺ solid tumors)	[103]
Implantable macrophage-derived vaccines	Macrophage-based vaccines	Prevents recurrence, boosts immune memory	Preclinical	[104]

These multifaceted strategies underscore the potential of macrophage-targeted therapy in overcoming immunotherapy

### Inhibition of macrophage-associated factors

Chemokine Antagonists: Adipose tissues in obesity secrete high levels of chemokines that attract macrophages to the tumor. Inhibitors of chemokines, such as CCL2 antagonists, can reduce TAM recruitment and attenuate tumor progression [95].

### Altering macrophage numbers and phenotypes

- Clodronate: A bisphosphonate that depletes macrophages, thereby reducing their tumor-promoting functions and leading to tumor regression [96].
- CSF1/CSF1R Inhibitors: Small molecules or monoclonal antibodies targeting CSF1 or its receptor can reduce M2 macrophages and promote M1 polarization, enhancing anti-tumor immunity [73].

### Neutralizing immunosuppression

- Immune Checkpoint Inhibitors: Antibodies targeting PD-1/PD-L1 can restore T cell activity by blocking TAM-mediated immunosuppression [97, 98]. However, TAM-mediated resistance through PD-L1 downregulation remains a challenge [92].
- PI3K-γ Inhibitors: These molecules activate NF-κB signaling in TAMs, promoting repolarization to the M1 phenotype and reducing PD-L1 expression, thereby sensitizing tumors to immunotherapy [99].
- Targeting Immunosuppressive Cytokines: Neutralization of IL-10 or TGF-β via antibodies or RNA interference can reverse immunosuppression and restore cytotoxic T cell function [100].

### Advanced therapeutic approaches

- Nanobiotechnology: Nanoparticles, including MOF-based systems, enable targeted drug delivery and M2-to-M1 repolarization. Inducing ferroptosis-related stress in TAMs is an innovative strategy to reprogram the TME [101, 102].
- CAR-M Therapy: Chimeric antigen receptor macrophages (CAR-Ms) represent an emerging immunotherapy, capable of repolarizing TAMs, inducing inflammation, and enhancing T-cell responses. Originally applied in ovarian cancer, CAR-M therapy shows promise for application in ORBC [103].
- Macrophage-Based Vaccines: Vaccination strategies utilizing macrophage-derived antigens have demonstrated potential in preventing tumor recurrence by promoting M1 macrophages infiltration and memory T cell activation. [104]

## Conclusion and outlook

Macrophages play a central and multifaceted role in ORBC, particularly in modulating inflammation, metabolic crosstalk, immune evasion, angiogenesis, and tumor microenvironment (TME) remodeling. Among them, adipose tissue macrophages (ATMs) and tumor-associated macrophages (TAMs) act as key intermediaries between obesity-induced metabolic dysfunction and breast cancer progression. TAMs in ORBC predominantly polarize into the M2 phenotype and secrete immunosuppressive cytokines (e.g.IL-10, TGF-β) and metabolites (e.g. lactate), fostering immune escape, neovascularization, and therapeutic resistance. The immunometabolic reprogramming between cancer cells and TAMs, especially through altered glycolysis and lipid metabolism, creates a self-reinforcing tumorigenic niche. This makes immunometabolism a critical axis in ORBC pathology and a potential intervention target.

Importantly, obesity influences the spatial heterogeneity and topological distribution of TAMs in the breast tumor microenvironment. Studies have shown that TAMs cluster in hypoxic and necrotic zones, perivascular niches, and invasive fronts, with their spatial location strongly correlating with functional polarization and clinical prognosis. Obesity-associated signals such as CCL2, CSF1, and leptin not only increase macrophages infiltration but also direct their spatial recruitment to metabolically stressed or angiogenic regions of the tumor. This spatial reprogramming complicates effective TAM targeting and suggests the necessity of spatial transcriptomic profiling in future research.

From a translational medicine perspective, macrophage-targeted therapies face several critical challenges. First, the heterogeneity and plasticity of TAMs make selective targeting difficult, especially given their dynamic phenotype shifts in response to environmental stimuli. Second, drug delivery remains a major barrier—systemically administered agents often fail to accumulate specifically in tumor-resident macrophages due to poor tissue penetration or off-target effects. Third, the preclinical models currently employed—mostly high-fat-diet murine models—may not fully recapitulate the complexity of human ORBC, particularly in terms of metabolic heterogeneity, comorbidities (e.g. diabetes, cardiovascular disease), and immune architecture. Thus, improved animal models and early-phase clinical trials are essential to validate findings in more clinically relevant settings.

In summary, macrophages are both pathogenic facilitators and promising therapeutic targets in ORBC. While strategies such as CSF-1R inhibition, immune checkpoint blockade, and nanotechnology-based interventions show potential in restoring immune function and inhibiting tumor progression, these approaches must be optimized to overcome TAM plasticity, spatial complexity, and delivery limitations. Future research should focus on dissecting obesity-induced immunometabolic programming, elucidating TAM spatial biology, and personalizing therapies based on obesity phenotype, metabolic state, and tumor subtype. Moreover, lifestyle interventions such as dietary regulation and physical activity, which modulate systemic inflammation and macrophages ‘ function, should be considered as adjuncts to enhance therapeutic outcomes. As highlighted by Mantovani et al. [57], a deeper understanding of macrophages plasticity and context-dependent behavior will be pivotal for developing next-generation macrophage-centered treatments in ORBC.

## Data Availability

No datasets were generated or analysed during the current study.
